# Severe heart failure due to peripartum cardiomyopathy

**DOI:** 10.1002/rcr2.1137

**Published:** 2023-04-09

**Authors:** Haruhiko Michimata, Toshiyuki Sumi, Daiki Nagayama, Yuta Koshino, Hiroki Watanabe, Yuichi Yamada, Hirofumi Chiba

**Affiliations:** ^1^ Department of Respiratory Medicine Hakodate Goryoukaku Hospital Hakodate Hokkaido Japan; ^2^ Department of Respiratory Medicine and Allergology Sapporo Medical University School of Medicine Sapporo Japan

**Keywords:** heart failure, peripartum cardiac tendinopathy, pulmonary oedema

## Abstract

Perinatal cardiomyopathy presents similarly to dilated cardiomyopathy and should be suspected in perinatal women presenting with dyspnoea, even with no previous history of heart disease.

## Graphical abstract

Perinatal cardiomyopathy develops during pregnancy or postpartum in women with no history of cardiac disease, presenting similarly to dilated cardiomyopathy. The pathogenesis is unknown but may be associated with both vascular damage and a genetic component. Physicians should consider pulmonary oedema due to perinatal cardiomyopathy in perinatal women with dyspnoea.

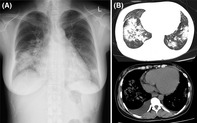

## CLINICAL IMAGE

**FIGURE 1:**
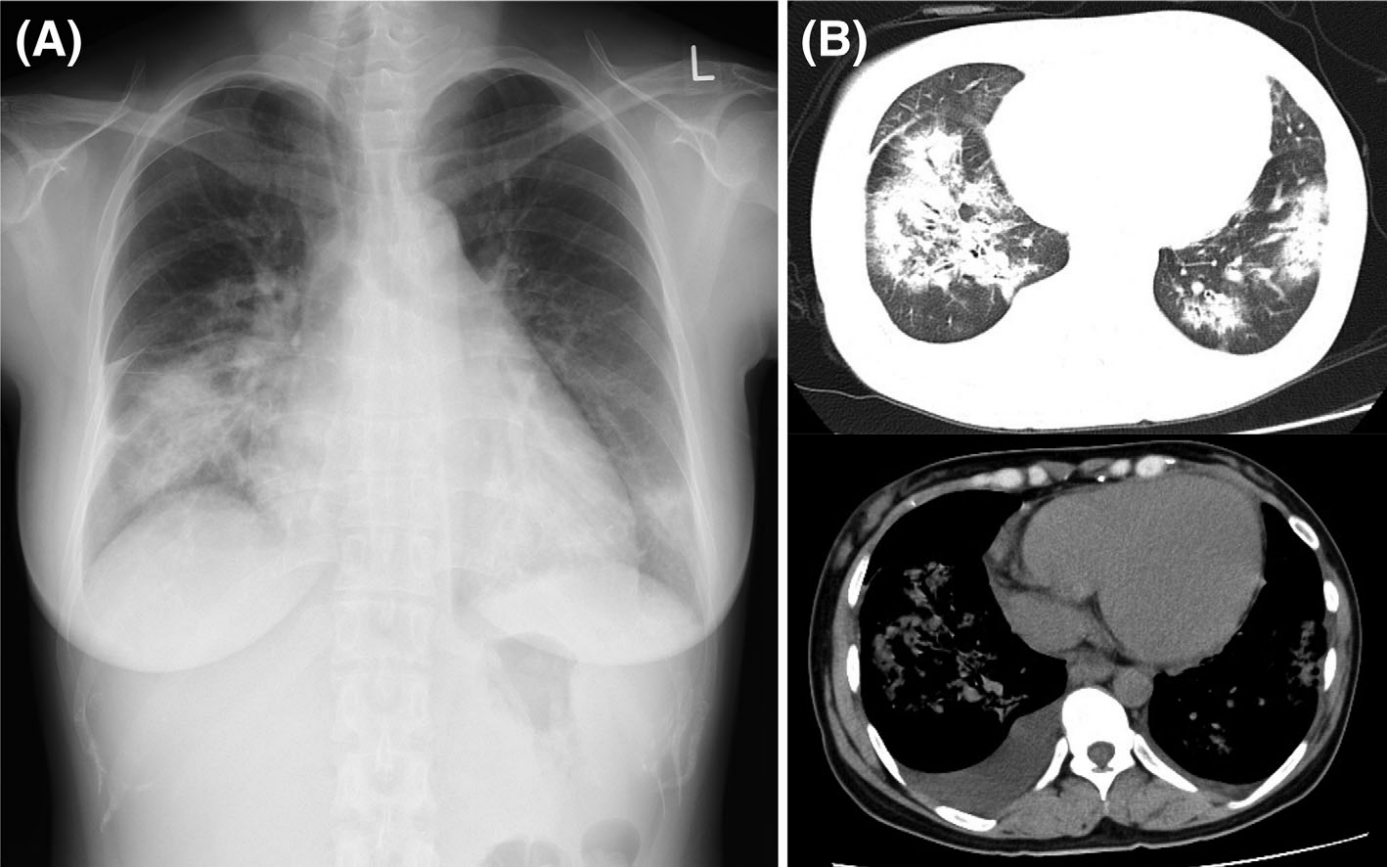
Imaging findings. (A) Chest radiograph showing remarkable cardiac enlargement and infiltrating shadows in the lung fields. (B) Chest computed tomography showing bilateral pulmonary consolidation and right pleural effusion

A 34‐year‐old woman, 2 months postpartum, presented after 1 week of dyspnoea. She had no history of cardiac disease; however, during pregnancy, she developed gestational hypertension and was treated with methyldopa hydrate, which stabilized her blood pressure during the full‐term delivery and was continued afterward. Initial chest radiography showed an enlarged heart (Figure 1A), and computed tomography showed right pleural effusion and bilateral infiltrating shadows (Figure 1B). Blood tests revealed elevated N‐terminal‐pro‐B‐type natriuretic peptide (NT‐ProBNP) at 6676 pg/mL (normal limit: ≤ 125). The right pleural effusion was transudative, and echocardiography revealed an extremely low ejection fraction (20%) and overall decreased wall motion. The patient was diagnosed with peripartum cardiomyopathy and was administered catecholamine.

Perinatal cardiomyopathy develops during pregnancy or postpartum in women with no history of cardiac disease, presenting similarly to dilated cardiomyopathy.^1^ The pathogenesis is unknown but may be associated with both vascular damage and a genetic component.^2^ Between 50% and 80% of women treated for perinatal cardiomyopathy recover near‐normal left ventricular systolic function (LVEF ≥50%) within 6 months.^2^ However, LVEF <30% at diagnosis is associated with low probability of left ventricular recovery, higher need for mechanical assistance or transplantation, and death.^2^ Physicians should consider pulmonary oedema due to perinatal cardiomyopathy in perinatal women with dyspnoea.

## AUTHOR CONTRIBUTIONS

*Conceptualization*: Toshiyuki Sumi. *Data curation*: Haruhiko Michimata and Daiki Nagayama. *Formal analysis*: Yuta Koshino and Hiroki Watanabe. *Investigation*: Yuichi Yamada. *Writing‐original draft*: Toshiyuki Sumi. *Writing‐review & editing*: Hirofumi Chiba.

## CONFLICT OF INTEREST STATEMENT

None declared.

## ETHICS STATEMENT

The authors declare that appropriate written informed consent was obtained for the publication of this manuscript and accompanying images.

## Data Availability

The data that support the findings of this study are available from the corresponding author upon reasonable request.
